# Prevalence and Population Genetics Analysis of *Enterocytozoon bieneusi* in Dairy Cattle in China

**DOI:** 10.3389/fmicb.2019.01399

**Published:** 2019-06-25

**Authors:** Hai-Yan Wang, Meng Qi, Ming-Fei Sun, Dong-Fang Li, Rong-Jun Wang, Su-Mei Zhang, Jin-Feng Zhao, Jun-Qiang Li, Zhao-Hui Cui, Yuan-Cai Chen, Fu-Chun Jian, Rui-Ping Xiang, Chang-Shen Ning, Long-Xian Zhang

**Affiliations:** ^1^Experimental and Research Center, Henan University of Animal Husbandry and Economy, Zhengzhou, China; ^2^College of Animal Science and Veterinary Medicine, Henan Agricultural University, Zhengzhou, China; ^3^College of Animal Science, Tarim University, Alar, China; ^4^Institute of Animal Health, Guangdong Academy of Agricultural Sciences, Guangzhou, China

**Keywords:** *Enterocytozoon bieneusi*, prevalence, dairy cattle, China, multilocus genotyping, zoonotic infection

## Abstract

*Enterocytozoon bieneusi*, an obligate intracellular pathogen, can infect various hosts. In this study, 3527 dairy cattle fecal specimens were collected from different geographic locations in China (including 673 from Shandong province, 1,440 from Guangdong province and 1,414 from Gansu province) and examined for the presence of *E. bieneusi* using polymerase chain reactions targeting the ribosomal internal transcribed spacer (ITS). The dominant genotypes identified were further subtyped by multilocus sequence typing. The overall prevalence of *E. bieneusi* was 14.2% (501/3527), with a significant difference in prevalence among the different geographical locations (*P* < 0.001). Our logistic regression analysis showed that all four variables (farming model, location, age, and clinical manifestations) had strong effects on the risk of contracting *E. bieneusi*. Sequence analysis revealed 11 genotypes: eight known genotypes (J, I, BEB4, BEB10, D, EbpC, CM19, and CM21) and three novel genotypes (named here as CGC1, CGC2, and CGC3). Genotypes J and I, the commonest, were found on all farms across the three provinces. Our linkage disequilibrium analysis showed a clonal population structure in the *E. bieneusi* dairy cattle population but the ITS genotypes had different population structures. Phylogenetic and haplotype network analysis showed the absence of geographical segregation in the *E. bieneusi* dairy cattle populations. Instead, they revealed the presence of host adaptation to the *E. bieneusi* populations in various animals. Our findings augment the current understanding of *E. bieneusi* transmission dynamics.

## Introduction

Microsporidia, a diverse group of emerging opportunistic pathogens with more than 1,300 named species, are classified as fungi (Mathis et al., 2005). Among approximately 17 human infective microsporidia species, *Enterocytozoon bieneusi* is the most commonly detected (Matos et al., 2012). *E. bieneusi* can cause gastrointestinal illnesses such as wasting syndrome and chronic diarrhea in immunocompromised people (organ transplant recipients, patients with cancer or AIDS), but remains asymptomatic in the immunocompetent (Didier and Weiss, 2011). *E. bieneusi* has also been detected in livestock, companion animals and wildlife, and even in environmental water samples (Santín and Fayer, 2011; Guo et al., 2014).

The DNA sequence of the ribosomal internal transcribed spacer (ITS) has been frequently used as the standard method for determining the genotypes of *E. bieneusi* (Santín and Fayer, 2009b), and phylogenetic analysis has revealed that over 300 *E. bieneusi* ITS genotypes cluster into at least 11 large groups (Li et al., 2018). Group 1 contains most genotypes found in humans (e.g., EbpC, D, EbpD, Peru8, Peru11, and type IV), and with its likely transmission between humans and other animals this group is considered to be zoonotic. Groups 2–11 have a narrow host range and only infect particular animals (e.g., ruminants, non-human primates, horses, and dogs) (Guo et al., 2014). To date, over 40 *E. bieneusi* genotypes have been identified in cattle worldwide, most of which belong to Group 2 (Fayer et al., 2003, 2007; Sulaiman et al., 2004; Santin et al., 2012; Del Coco et al., 2014; Ma et al., 2015; Zhao et al., 2015; da Silva Fiuza et al., 2016; Li J. et al., 2016; Wang et al., 2016; Zhang et al., 2018). However, some genotypes (I, J, BEB4, and BEB6) from Group 2, which were originally regarded as ruminant-specific, are considered to have reduced host specificity because of the sporadic infections they cause in other hosts including humans (Sak et al., 2011; Zhang et al., 2011; Wang et al., 2013; Jiang et al., 2015), implying the possibility of them having zoonotic transmission.

Nevertheless, the use of a single-marker typing method has limitations in representing the whole genome of *E. bieneusi* (∼ 6 Mb total length), and with the possibility of a sexual phase in the *E. bieneusi* lifecycle (Widmer and Akiyoshi, 2010) such an approach will be inadequate for inferring subgroup-level phylogenies. The multilocus sequence typing (MLST) tool approach, which has higher resolution, has been effectively used to characterize the population genetic structures of *Cryptosporidium*, *E. bieneusi*, and *Cyclospora cayetanensis* (Feng et al., 2011; Li et al., 2012, 2013; 2017; Karim et al., 2014; Guo et al., 2016; Wan et al., 2016). Geographical regions, transmission intensities, genetic variation and adaptive selection within species contribute to shape diverse population structures: clonal, epidemic, and panmictic (Tanriverdi et al., 2006). These evoluting processes have caused the association of the population structures with specific transmission patterns, parasite virulence, the emergence of host-adapted and geographical segregation and hypertransmissible populations with different genetic structures and public health potential (Feng et al., 2018).

As one of the largest animal husbandry countries, China raises very large numbers of dairy cattle annually. Although many studies have reported the prevalence and genotypes of *E. bieneusi* in dairy cattle from different Chinese provinces or cities, including Henan, Hebei, Tianjin, Ningxia, and Xinjiang (Li J. et al., 2016; Wang et al., 2016; Hu et al., 2017; Qi et al., 2017), these observations on *E. bieneusi* in China would benefit from substantiation by studies conducted in other geographic locations to fully determine the overall picture. Therefore, in this study, we investigated the prevalence of *E. bieneusi* in dairy cattle from other geographic locations in China and assessed the population structure of the common *E. bieneusi* ITS genotypes by MLST analyses.

## Materials and Methods

### Ethics Statement

This study was performed strictly according to the recommendations of the Guide for the Care and Use of Laboratory Animals of the Ministry of Health, China. Our protocol was reviewed and approved by the Research Ethics Committee (Approval No. LVRIAEC 2016-011) of Henan Agricultural University (Zhengzhou city, China). The locations where we sampled did not involve endangered or protected species and no specific permits were required. All fecal specimens were collected based on the accessibility of the animals for sampling and each owner’s willingness to participate in the study.

### Collection of Fecal Samples and DNA Extraction

In total, 3527 feces samples from dairy cattle under 1 year of age from 24 farms in Shandong (673 samples from 5 farms), Guangdong (1,440 samples from 10 farms), and Gansu province (1,414 samples from 9 farms) were collected and examined. Each dairy farm was sampled on one occasion between November 2017 and September 2018, and 15–20% of the each herd was sampled. The fecal samples were collected from the rectum or immediately picked up using sterile gloves after defecation, and then stored on ice. Genomic DNA was extracted from each sample using the E.Z.N.A.^®^ Stool DNA Kit (D4015-02, Omega Bio-Tek, Inc., Norcross, GA, United States) according to the manufacturer’s instructions and then stored at -20°C until used for polymerase chain reaction (PCR) analyses. All samples were processed within 24 h of collection.

### PCR Amplification

*Enterocytozoon bieneusi* genotypes from the dairy cattle residing in different geographical regions were determined by nested PCR amplification of an ∼390 bp fragment of the ribosomal ITS spacer, and using primers whose sequences have been described previously (Buckholt et al., 2002) (Table 1). Each PCR was conducted in a 25 μL volume, containing 0.3 μM of each primer, 12.5 μL 2 × EasyTaq PCR SuperMix (TransGen Biotech Co., Ltd., Beijing, China), 1 μL of genomic DNA for the primary PCR and 1 μL of the primary amplification product for the secondary PCR, and 10.9 μL of deionized water. Positive (dairy cattle-derived genotype J DNA) and negative controls (sterile water) were included in each test.

**Table 1:** PCR primers used in this study.

Gene locus	Primer	Sequence (5′–3′)	Amplicon length (bp) (GenBank accession number)	Annealing temperature (°C)
ITS	EBITS3	GGTCATAGGGATGAAGAG	435	57
	BITS4	TTCGAGTTCTTTCGCGCTC		
	BITS1	GCTCTGAATATCTATGGCT	390 (MK559494–MK559496)	55
	EBITS2.4	ATCGCCGACGGATCCAAGTG		
MS1	F1	CAAGTTGCAAGTTCAGTGTTTGAA	843	58
	R1	GATGAATATGCATCCATTGATGTT		
	F2	TTGTAAATCGACCAAATGTGCTAT	676 (MH560534–MH560563)	58
	R2	GGACATAAACCACTAATTAATGTAAC		
MS3	F1	CAAGCACTGTGGTTACTGTT	702	55
	R1	AAGTTAGGGCATTTAATAAAATTA		
	F2	GTTCAAGTA ATTGATACCAGTCT	537 (MH560519–MH560526)	55
	R2	CTCATTGAATCTAAATGTGTATAA		
MS4	F1	GCATATCGTCTCATAGGAACA	965	55
	R1	GTTCATGGTTATTAATTCCAGAA		
	F2	CGAAGTGTACTACATGTCTCT	885 (MH560527–MH560533)	55
	R2	GGACTTTAATAAGTTACCTATAGT		
MS7	F1	GTTGATCGTCCAGATGGAATT	684	55
	R1	GACTATCAGTATTACTGATTATAT		
	F2	CAATAGTAAAGGAAGATGGTCA	471 (MH560564–MH560566)	55
	R2	CGTCGCTTTGTTTCATAATCTT		


### MLST PCR and Sequencing

**FIGURE 1:**
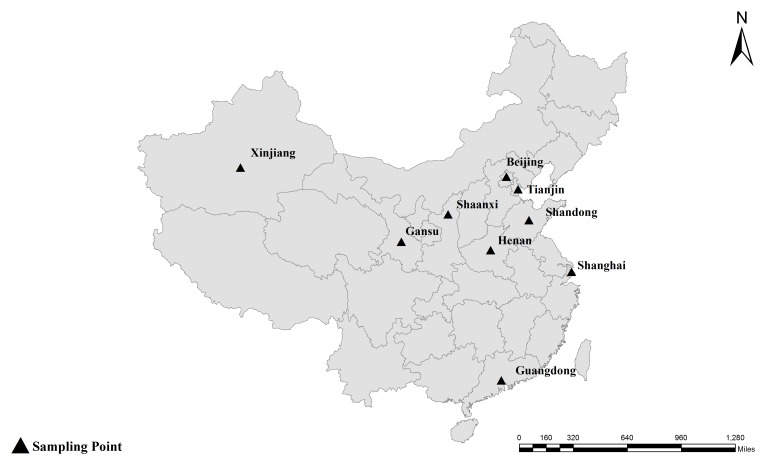
Specific locations where the samples were collected in this study. Locations ▴.

Together with the ITS, one minisatellite (MS4) and three microsatellite markers (MS1, MS3, and MS7) were used in the MLST analysis. The PCR primers and amplification conditions used for the four markers were the same as those previously described (Feng et al., 2011) (Table 1). Considering *E. bieneusi* ITS genotypes J, I, and BEB4 as being the dominant genotypes found in different geographic locations in China and their recently certified potential zoonotic properties, specimens that belonged to these genotypes were selected for further MLST analysis. Specifically, 2–3 isolates were chosen from each *E. bieneusi*-positive farm in the different geographical locations (Figure 1). With these selected samples, we tried to incorporate representatives of the different dominant pathogen genotypes, and representatives of the different clinical signs and age groups for the dairy cattle. A total of 155 *E. bieneusi* specimens were used in this study. The number of isolates and their ITS genotype designations by geographic location are shown in Table 2. Most of the specimens were genotyped in this study, whereas the remaining specimens from Shaanxi and Shanghai were included and genotyped by the same technique in previous studies (Wang et al., 2016; Tang et al., 2018).

**Table 2:** The *E. bieneus*i isolates used for further intra-genotypic variation analysis in this study and their associated ITS genotypes.

Location	Number	ITS genotype (number)	References
Beijing	14	J (5); I (4); BEB4 (5)	Hu et al., 2017
Tianjin	15	J (11); BEB4 (4)	Hu et al., 2017
Henan	20	J (11); I (5), BEB4 (4)	Li J. et al., 2016
Guangdong	23	J (12); I (8); BEB4 (3)	This study
Shandong	21	J (18); I (1); BEB4 (2)	This study
Gansu	21	J (8); I (8); BEB4 (5)	This study
Xinjiang	21	J (4); I (9); BEB4 (8)	Qi et al., 2017
Shaanxi	10	J (5); I (5)	Wang et al., 2016
Shanghai	10	J (8); BEB4 (2)	Tang et al., 2018
Total	155	J (82); I (40); BEB4 (33)	


Secondary PCR products were agarose gel electrophoresed, and then visualized and examined after GelRed^TM^ (Biotium Inc., Hayward, CA, United States) staining. The secondary PCR products were bidirectionally sequenced on an ABI PRISM^TM^ 3730 XL DNA Analyzer using the BigDye Terminator v3.1 Cycle Sequencing Kit (Applied Biosystems, Foster City, CA, United States). Raw sequences were assembled and edited with Chromas Pro version 2.1.3 (Technelysium Pty., Ltd., Helensvale, QLD, Australia). The sequences obtained were compared with the reference sequences downloaded from the National Center for Biotechnology Information^1^ using Clustal X 2.0^2^.

### Linkage Disequilibrium (LD) Analysis

The values for the standardized index of association (*I*^S^_A_) were calculated using the LIAN 3.5 program^3^ on the five-locus haplotypes. Moreover, the variance of pair-wise differences (*V*_D_) and 95% confidence limits (*L*) were also calculated as another test of LD.

### Phylogenetic and Sub-Population Analysis

An analysis of the phylogenetic relationships among the *E. bieneusi* isolates was performed by ITS sequencing and the resultant sequences were concatenated for all 5 polymorphic markers by a neighbor-joining (NJ) analysis in MEGA 7.01, as based on the Kimura 2-parameter model using 1000 bootstrap replicates. Additionally, median-joining phylogenies were generated using Network software version 5.0 ^4^ under the default parameters. Networks were then arranged by hand and nodes colored using Network Publisher version 5.0.0.0^5^. *E. bieneusi* isolates from different hosts and geographic origins, including the *E. bieneusi* isolates belonging to ITS zoonotic Group 1 (genotypes D, EbpC, type IV, horse1, Nig2, EBITS3, Henan-1, WL11, CM1, and CM2), Group 6 (genotypes horse2 and Nig3), Group 10 (genotypes CHB1, CSK1, and ABB1) and an outlier genotype (Nig5) were also included in the multilocus phylogeny and haplotype network analysis (Karim et al., 2014; Wan et al., 2016; Li et al., 2019).

### Statistical Analysis

Comparisons of *E. bieneusi* prevalence (*ó*) in dairy cattle between the different geographical locations (*x*1), ages (*x*2), clinical signs in the animals (*x*3), and farming model (*x*4) were performed using the chi-squared test. All results were considered statistically significant at *P* < 0.01. Odds ratios (ORs) and 95% confidence intervals (95% CIs) were calculated to explore the strength of the association between *E. bieneusi* positivity and the variables tested. The impacts of the multiple variables were also evaluated by multivariable regression analysis using SPSS 22.0 version (SPSS Inc., Chicago, IL, United States).

## Results

### Prevalence of *E. bieneusi*

Of the 3,527 dairy cattle fecal specimens we tested, 501 (14.2%) were found to be *E. bieneusi*-positive by nested PCR amplification of the ITS region. *E. bieneusi* was detected in 21 of the 24 farms surveyed, with infection rates ranging between 0 and 42.4% (Table 3). The results of the univariate and multiple analyses are summarized in Table 4. In the final model, all four variables had strong effects on *E. bieneusi* prevalence, as described by the equation *y* = -0.455 ×*x*4 + 2.069 ×*x*1 + 0.929 ×*x*2 + 0.970 ×*x*3 - 4.124. Farming model had a negative effect on the risk of *E. bieneusi*, for which the OR was 0.65 (95% CI 0.52–0.82). Dairy cattle managed by outdoor-free practices (18.6% positive) showed a significantly higher *E. bieneusi* prevalence compared with those managed by intensive-closed practices (13.1% positive). Notably, clinical manifestations in the cattle, age and the geographical region also had strong effects on the risk of contracting *E. bieneusi*. Dairy cattle in Gansu Province (22.6% positive) were considered to have higher positivity rates compared with those from the two other provinces (Guangdong, 11.1%; Shandong, 3.1%). Furthermore, post-weaned (15.9% positive) dairy cattles and diarrheal animals (21.1% positive) were more susceptible to *E. bieneusi* than pre-weaned cattles (9.1% positive) and animals with clinical signs (13.5% positive), respectively, for which the ORs were 2.98 (95% CI 2.28–3.89) and 3.19 (95% CI 2.32–4.38), respectively.

**Table 3:** *Enterocytozoon bieneusi* genotypes identified in dairy cattle from different farms in three Chinese geographic regions.

Location	Farm ID	Farming model	No. tested	No. positive (%)	Genotype (n)
Shandong Province	1	Intensive-closed	141	5 (3.54%)	J (5)
	2	Intensive-closed	136	0	–
	3	Intensive-closed	121	6 (4.41%)	BEB4 (1), J (5)
	4	Intensive-closed	135	3 (2.22)	J (3)
	5	Outdoor-free	140	7 (5.0%)	J (5), I (1), BEB4 (1)
Subtotal			673	21 (3.12)	J (18), BEB4 (2), I (1)
Guangdong Province	1	Intensive-closed	82	6 (7.3%)	J (6)
	2	Outdoor-free	67	9 (13.4%)	I (9)
	3	Outdoor-free	40	0	–
	4	Intensive-closed	111	25 (22.5%)	I (7), J (14), D (4)
	5	Outdoor-free	138	23 (16.7%)	I (9), J (13), BEB4 (1)
	6	Intensive-closed	118	17 (14.4%)	J (13), BEB4 (2), EbpC (2)
	7	Intensive-closed	374	28 (7.4%)	I (18), J (10)
	8	Outdoor-free	12	0	–
	9	Intensive-closed	319	27 (8.4%)	I (23), J (4)
	10	Intensive-closed	179	25 (13.9%)	I (25)
Subtotal			1440	160 (11.1%)	J (60), I (91), BEB4 (3), EbpC (2), D (4)
Gansu Province	1	Intensive-closed	125	30 (24)	J (20), I (10)
	2	Intensive-closed	55	8 (14.5%)	I (8)
	3	Intensive-closed	94	22 (23.4%)	J (18), I (4)
	4	Outdoor-free	125	53 (42.4%)	J (30), I (6), CGC 3^a^ (11), BEB4 (5), CM21 (1)
	5	Outdoor-free	137	31 (22.6%)	J (15), I (7), CGC 1^a^ (6), BEB10 (3)
	6	Outdoor-free	57	10 (17.5%)	J (9), I (1)
	7	Intensive-closed	226	8 (3.5%)	J (8)
	8	Intensive-closed	200	4 (2%)	J (4)
	9	Intensive-closed	395	154 (40%)	J (43), I (98), CGC 2^a^ (8), CM19 (5)
Subtotal			1414	320 (22.6%)	J (155), I (126), BEB4 (5), BEB10 (3), CM19 (5), CM21 (1), CGC1 (6)^a^, CGC2 (8)^a^, CGC3 (11)^a^
Total	24		3527	01 (14.2)	J (225), I (226), BEB4 (10), BEB10 (3), D (4), EbpC (2), CM19 (5), CM21 (1), CGC1 (6)^a^, CGC2 (8)^a^, CGC3 (11)^a^


*^a^Novel types from this study.*

**Table 4:** Factors associated with the prevalence of *E. bieneusi* in dairy cattle in three Chinese geographic regions.

Factor	Category	No. tested	No. positive	% (95% CI)	OR (95% CI)^a^	*P*-value^a^	OR (95% CI)^b^	*P*-value^b^
Location	Shandong Province	673	21	3.1 (1.8–4.4)	Reference	*P <* 0.01	Reference	*P <* 0.01
	Guangdong Province	1440	160	11.1 (9.5–12.7)	3.88 (2.44–6.18)		4.77 (2.97–7.65)	
	Gansu Province	1441	320	22.6 (20.4–24.8)	9.08 (5.78–14.27)		13.20 (8.28–21.04)	
Age	Pre-weaned	897	82	9.1 (7.3–11.0)	Reference	*P* < 0.01	Reference	*P* < 0.01
	Post-weaned	2630	419	15.9 (14.5–17.3)	1.88 (1.47–2.42)		2.98 (2.28–3.89)	
Clinical symptom	Non-diarrhea	3196	431	13.5 (12.3–14.7)	Reference	*P* < 0.01	Reference	*P* < 0.01
	Diarrhea	331	70	21.1 (16.7–25.6)	1.72 (1.30–2.28)		3.19 (2.32–4.38)	
Farming model	Outdoor-free	716	133	18.6 (18.0–19.2)	Reference	*P* < 0.01	Reference	*P* < 0.01
	Intensive-closed	2811	368	13.1 (12.9–13.3)	0.66 (0.53–0.82)		0.65 (0.52–0.82)	
Total		3527	501	14.2 (13.1–15.4)				


*^a^Univariate analysis; ^b^Multivariate analysis.*

### Genotype Distribution

**FIGURE 2:**
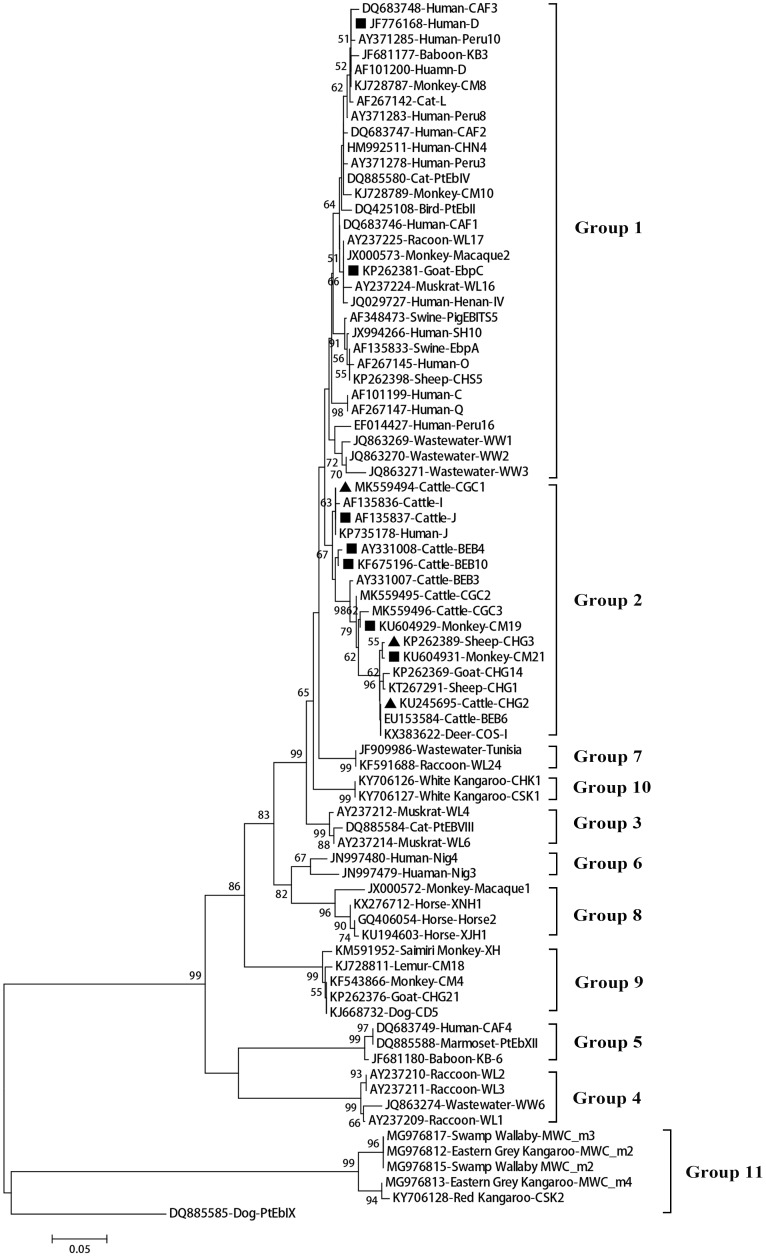
Phylogenetic relationships among the *E. bieneusi* genotypes identified in this study and other reported genotypes. The phylogeny was inferred from the Neighbor-joining (NJ) analysis of the ITS sequences based on the distances calculated using the Kimura 2-parameter model. Bootstrap values of *N* > 50% from 1,000 replicates are shown at the nodes. Known and new genotypes are indicated by hollow and filled triangles, respectively.

A total of 11 *E. bieneusi* ITS genotypes were identified from the 501 successfully sequenced specimens, including eight known genotypes (J, I, BEB4, BEB10, D, EbpC, CM19, and CM21) and three novel genotypes (CGC1, CGC2, and CGC3). Among them, *E. bieneusi* ITS genotypes J (*n* = 225, 44.9%) and I (*n* = 226, 45.1%) were the dominant ones in our study (Table 3). The remaining genotypes were seen in only 0–10 *E. bieneusi*-positive calves. Two genotypes (D and EbpC) clustered into zoonotic Group 1, while the remaining genotypes clustered into Group 2 (Figure 2). Among the three geographic locations, Gansu province showed the highest genetic diversity in its sampled cattle (nine *E. bieneusi* ITS genotypes) compared with Guangdong province (with five genotypes) and Shandong province (with three genotypes).

### Multilocus Sequence Typing (MLST) and Analysis

Altogether, 106 specimens were successfully amplified at all five loci, generating 30, 8, 7, and 3 genotypes at MS1, MS3, MS4, and MS7 loci, respectively. A total of 71 multilocus genotypes (MLGs) were formed (Supplementary Table S1). The *I*^S^_A_ values for the overall dairy cattle population and ITS genotype subpopulations are shown in Table 5. When all the isolates were used in the analysis, *I*^S^_A_ was >0 and *V*_D_ was greater than L, indicating the presence of LD and a clonal population structure for *E. bieneusi* in the overall dairy cattle population of China. Considering each group of isolates with the same MLST subtype as one individual, the *I*^S^_A_ value obtained was still above zero for the overall dairy cattle population (*I*^S^_A_ = 0.0513, *V*_D_ > *L*) and ITS genotype J (*I*^S^_A_ = 0.0917, *V*_D_ > *L*), suggesting a clonal population structure. In contrast, evidence for linkage equilibrium (LE) was obtained for ITS genotype I and BEB4 (genotypes I: *I*^S^_A_ = 0.0394, *P*_MC_ = 0.0134, and *V*_D_ < *L*; genotypes BEB4: *I*^S^_A_ = 0.0484, *P*_MC_ = 0.0079, and *V*_D_ < *L*), finally indicating that the two subpopulations under comparison had epidemic population structures.

**Table 5:** Results of the linkage disequilibrium analysis based on the allelic profile data from five genetic loci.

Population	No.	Hd	*I*^S^_A_	*P*_MC_	*V*_D_	*L*	*V*_D_ > L
All (10 locations)	106	0.6386 ± 0.1449	0.1126	<0.001	1.0649	0.7673	Y
All (10 locations)^a^	71	0.9392 ± 0.1505	0.0513	<0.001	0.8442	0.7472	Y
I	32	0.4303 ± 0.1550	0.1154	<0.001	1.0890	0.8916	Y
J	43	0.4901 ± 0.1967	0.1064	<0.001	0.6780	0.5228	Y
BEB4	31	0.4413 ± 0.1562	0.2100	<0.001	1.3711	0.8797	Y
I^a^	22	0.4877 ± 0.1687	0.0394	0.0134	0.7869	0.8583	N
J^a^	32	0.5015 ± 0.1989	0.0917	<0.001	0.6275	0.5130	Y
BEB4^a^	17	0.4926 ± 0.1861	0.0484	0.0079	0.6653	0.6801	N


*Hd, mean genetic diversity; I^*S*^_*A*_, standardized index of association calculated using the LIAN 3.5 program; P_*MC*_, significance of obtaining this value in 1,000 simulations using the Monte Carlo method; V_*D*_, variance of pairwise differences; L, 95% critical value for V_*D*_; V_*D*_ > L indicates linkage disequilibrium.^*a*^Considering isolates with the same MLG as one individual*.

### Phylogenetic and Structure Analysis

**FIGURE 3:**
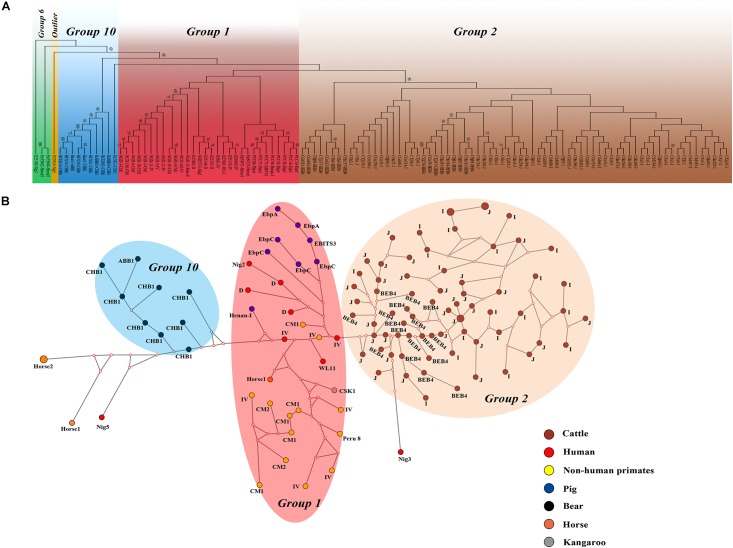
Phylogenetic and haplotype network of *E. bieneusi* isolates from different hosts and geographic origins. All dairy cattle *E. bieneusi* isolates (*n* = 71) identified in this study were included in the analyses and some of the Group 1 isolates (*n* = 30) with genotypes being D, EbpC, type IV, horse1, Nig2, EBITS3, Henan-1, WL11, CM1, and CM2, Group 6 isolates (*n* = 3) with genotypes being horse2 and Nig3, Group 10 isolates (*n* = 10) with genotypes being CHB1, CSK1, and ABB1, and an outlier isolate genotype Nig5 were selected for comparative analysis. **(A)** NJ phylogenetic analysis of all *E. bieneusi* isolates by the Kimura 2-parameter model, implemented in MEGA version 7.01. The letters B, H, Ho, P, C, N, and K, indicate the isolates were sampled from bear, human, horse, pig, cattle, non-human primates, and kangaroo, respectively. Bootstrap values of *N* > 50% from 1,000 replicates are displayed. **(B)** Median-joining analysis of the MLST data from all *E. bieneusi* specimens using the Network program. Circles are proportional to the frequency of each genotype and node sizes are proportional to the total haplotype frequencies. The colors within the circles represent the different ITS genotypes.

We performed multilocus phylogenetic and genetic network analyses for the *E. bieneusi* isolates from dairy cattle (*n* = 71) and other hosts (pigs, horse, humans, non-human primates, bears, and kangaroo, *n* = 44). All specimens used in the phylogenetic analysis formed three main clusters; one contained zoonotic MLGs from NHPs, humans and pigs, the remaining two contained MLGs with Group 2 and Group 10 that are specific to cattles and bears, respectively. No clear geographically segregated groups were seen among all the dairy cattle specimens (Figure 3). Median-joining network analysis showed the zoonotic MLGs were in the central position, while isolates from dairy cattles and bears occupied the peripheral position.

## Discussion

In the present study, the overall infection rate for *E. bieneusi* was 14.2% (501/3527). Different infection rates in dairy cattle have been reported for studies from China, including Henan and Ningxia (29.3%), Hebei and Tianjin (19.4%), Shaanxi (19.5%), Xinjiang (17.7%), and Shanghai (26.5%) (Li J. et al., 2016; Wang et al., 2016; Hu et al., 2017; Qi et al., 2017; Tang et al., 2018), and for North America (17.0 and 24.0%) (Fayer et al., 2003; Santín and Fayer, 2009b), Brazil (17.5%) (da Silva Fiuza et al., 2016), Argentina (14.3%) (Del Coco et al., 2014), and the Czechia (2.5%) (Juránková et al., 2013). The discrepant values among these studies might result from various factors, such as differences in the animal management systems, sample sizes, climatic and environmental conditions, as well as the health status of the animals. In our analysis of the effects of multiple variables on *E. bieneusi*, post-weaned dairy cattles were more susceptible to *E. bieneusi* than pre-weaned animals, which agreed with a previous study in Maryland where pre-weaned calves (11.7%) showed a significantly lower prevalence than post-weaned calves (44.4%) (Santín and Fayer, 2009a). Moreover, the farming management system showed a strong effect on the risk of contracting an *E. bieneusi* infection, probably because free-range dairy cattle have more opportunities to come into contact with contaminated food and water than those kept indoors.

Sequence analysis of the ITS sequences from the *E. bieneusi*-positive isolates highlighted the presence of high genetic diversity in the *E. bieneusi* genotypes. We identified genotypes I and J as the dominant ones in the present study. Similar results to ours have been reported in the Czechia, United States, Brazil, Argentina, and China (Sulaiman et al., 2004; Fayer et al., 2007; Santin et al., 2012; Juránková et al., 2013; Del Coco et al., 2014; Jiang et al., 2015; Ma et al., 2015; Zhao et al., 2015; da Silva Fiuza et al., 2016; Li J. et al., 2016; Wang et al., 2016). These dominant genotypes (notably I, J, BEB4, and BEB6), which were previously considered to be adapted to ruminants, can have a broad-host range and are therefore becoming of increasing zoonotic concern. For example, genotype I was detected in monkeys (Karim et al., 2014), and genotype J in deer (Santín and Fayer, 2015), chickens (Reetz et al., 2002), and pigeons (Pirestani et al., 2013), while BEB4 was detected in pigs, and BEB6 in deer (Zhao et al., 2014), horses (Qi et al., 2016), and pet chinchillas (Qi et al., 2015). The common *E. bieneusi* genotypes have also been reported in children in China and in immunocompetent people in the Czechia (Sak et al., 2011; Zhang et al., 2011; Wang et al., 2013; Jiang et al., 2015). The occurrence of zoonotic genotypes suggests that dairy cattle may be potential reservoirs of infection and play a role in the epidemiology of *E. bieneusi*.

In the present study, strong LD has revealed a clonal population structure in the overall population of dairy cattle, further supporting the finding that *E. bieneusi* undergoes predominantly clonal propagation in dairy cattle, which probably reflects the narrow-host range and lower transmission intensity of *E. bieneusi* genotypes. This observation is similar to what has been reported elsewhere; specifically that *E. bieneus*i isolates from AIDS patients in Peru, Nigeria, and India have a clonal population structure (Li et al., 2013). A clonal population structure for *E. bieneusi* was also found in non-human primates, pigs, pandas, and fur animals (Karim et al., 2014; Li W. et al., 2016, 2017; Wan et al., 2016). Nevertheless, the possibility of genetic recombination among some of the genotypes cannot be excluded. Our analysis of the allelic profile data showed that ITS genotypes I and BEB4 are in LE and have epidemic population structures (*I*^S^_A_ was >0 and *V*_D_ < *L*), which indicates that genetic recombination has occurred among them. This situation is similar to that reported previously where a clonal population was reported for the zoonotic ITS genotypes D and Type IV and an epidemic population in genotype A, which only infects human AIDS patients (Li et al., 2012, 2013). Further information was reported by Karim et al. (2014) who observed that the ITS genotypes CM1, Type IV and D had epidemic population structures, while Li W. et al. (2016) revealed population differentiation in fur animals with ITS genotype D from two known human *E. bieneusi* populations with ITS genotypes D and IV (a clonal structure) and with ITS genotype A (an epidemic structure) in their study. Collectively, the observations on the overall clonality and epidemic population structures of sub-population structures in dairy cattle, AIDS patients in Peru, Nigeria, and India, non-human primates, and fur animals further support the probability of sexual recombination occurring in *E. bieneusi* (Lee et al., 2008). The determination of the population genetic structure of *E. bieneusi* is undoubtedly vital to the understanding of its transmission patterns.

The high MLG diversity revealed in the present study on *E. bieneusi* is based on only three ITS genotypes (I, J, and BEB4). The *E. bieneusi* MLGs from dairy cattle showed no signs of geographical segregation by phylogenetic and haplotype networks analysis, indicating that they most likely originate from a single clonal type. Although this phenomenon might be related to frequent animal transport, the food and feed trade, and frequent floods, the *E. bieneusi* MLST data from different hosts shows a clear host separation, revealing the potential occurrence of directed genetic differentiation from zoonotic to host-adapted in *E. bieneusi* (Li and Xiao, 2019; Li et al., 2019). More MLST data are patently needed to further assess the level of host specificity for this species.

## Conclusion

*Enterocytozoon bieneusi* in dairy cattle in China exhibits a high level of genetic diversity in our study. The detection of *E. bieneusi* zoonotic genotypes suggests that dairy cattle may be reservoir hosts for zoonotic *E. bieneusi* infections and play a role in the epidemiology of this fungal pathogen. MLST analysis revealed a high level of MLG diversity in the same ITS gene sequences. LD analysis revealed a clonal structure within the overall population of dairy cattle. No significant geographic segregation in *E. bieneusi* MLGs from dairy cattle was observed. Instead, the data have revealed the presence of host adaptation of *E. bieneusi* to different hosts. The findings presented here enhance our current understanding of the transmission dynamics of this pathogen.

## Data Availability

The data sets supporting the conclusions of this article are included in the article. All ITS, MS1, MS3, MS4, and MS7 nucleotide sequences from dairy cattle-isolated *E. bieneusi* in this study are deposited in the GenBank database under accession numbers MK559494–MK559496, MH560534–MH560563, MH560519–MH560526, MH560527–MH560533, and MH560564–MH560566, respectively.

## Author Contributions

L-XZ conceived and designed the research. M-FS, Z-HC, and MQ collected the samples. H-YW, J-FZ, S-MZ, J-QL, Y-CC, and MQ performed the experiments. D-FL, R-JW, F-CJ, R-PX, and C-SN analyzed the data. H-YW wrote the manuscript. All authors read and approved the final manuscript.

## Conflict of Interest Statement

The authors declare that the research was conducted in the absence of any commercial or financial relationships that could be construed as a potential conflict of interest.

## Footnotes

1 https://www.ncbi.nlm.nih.gov/

2 http://www.clustal.org

3 guanine.evolbio.mpg.de/cgi-bin/lian/lian.cgi.pl

4 www.fluxus-engineering.com/sharenet.htm

5 http://fluxus-engineering.com/nwpub.htm
